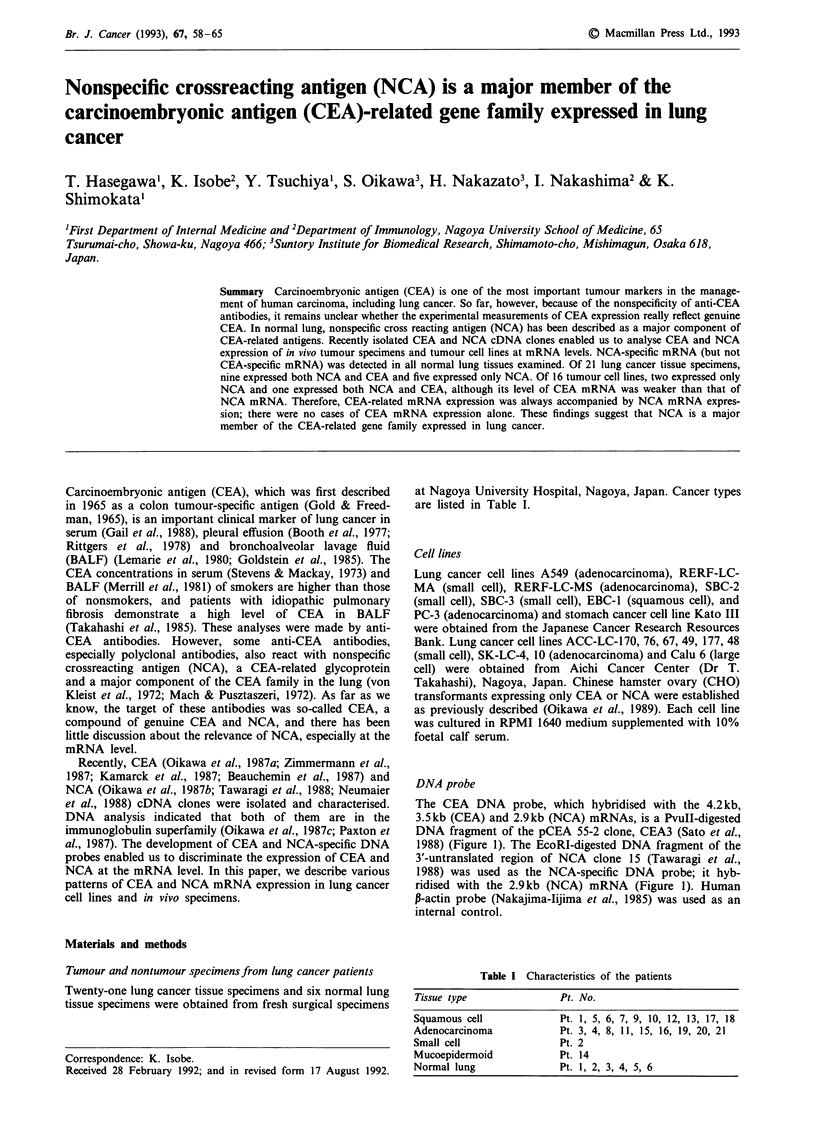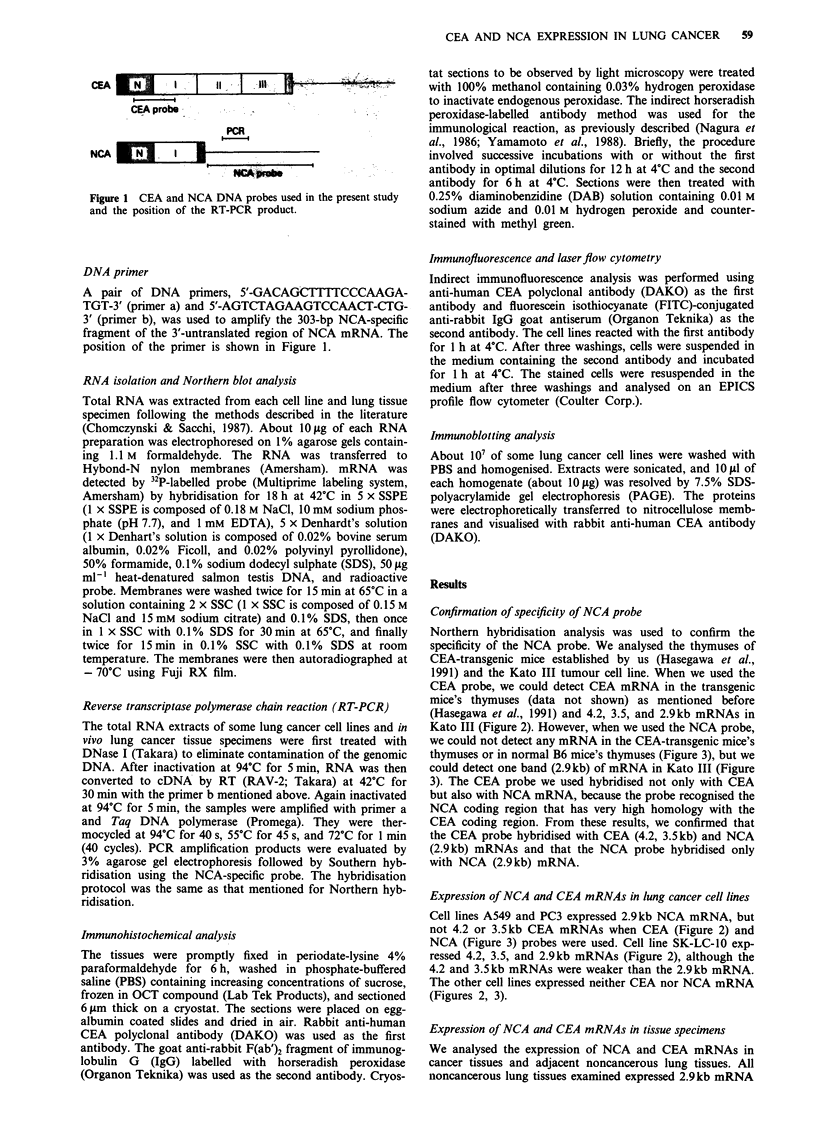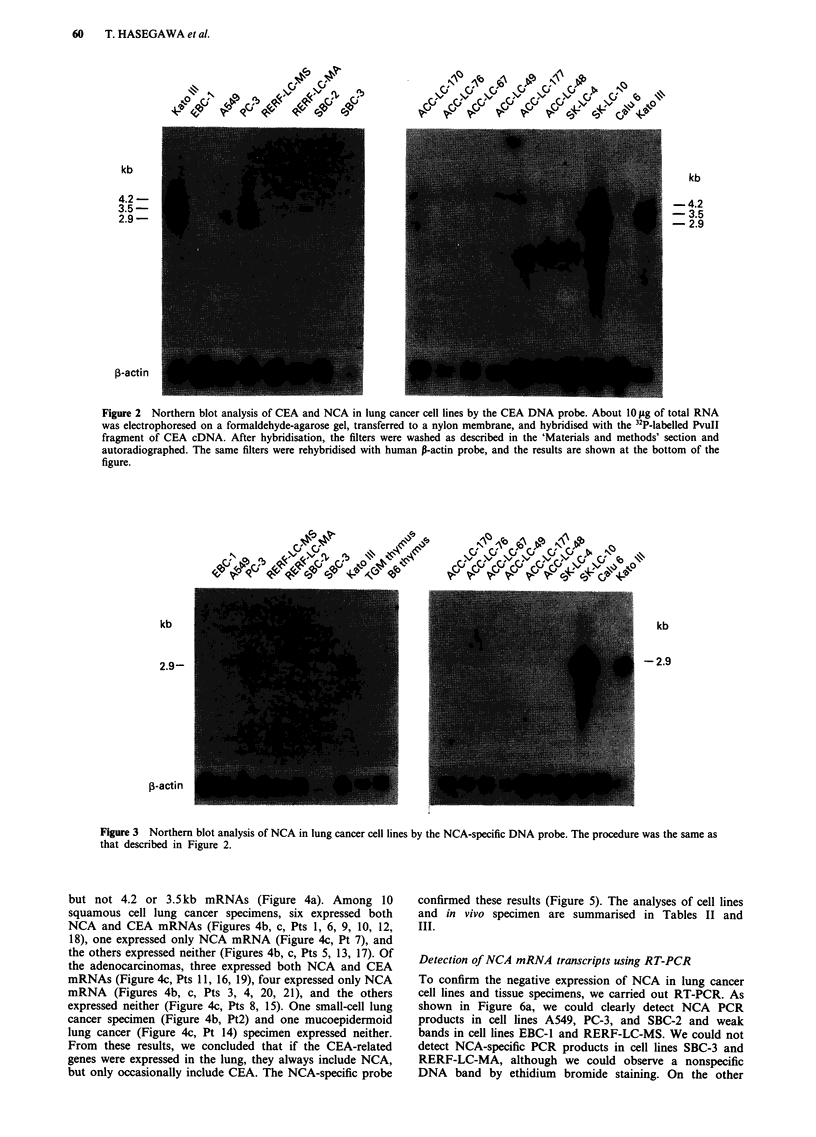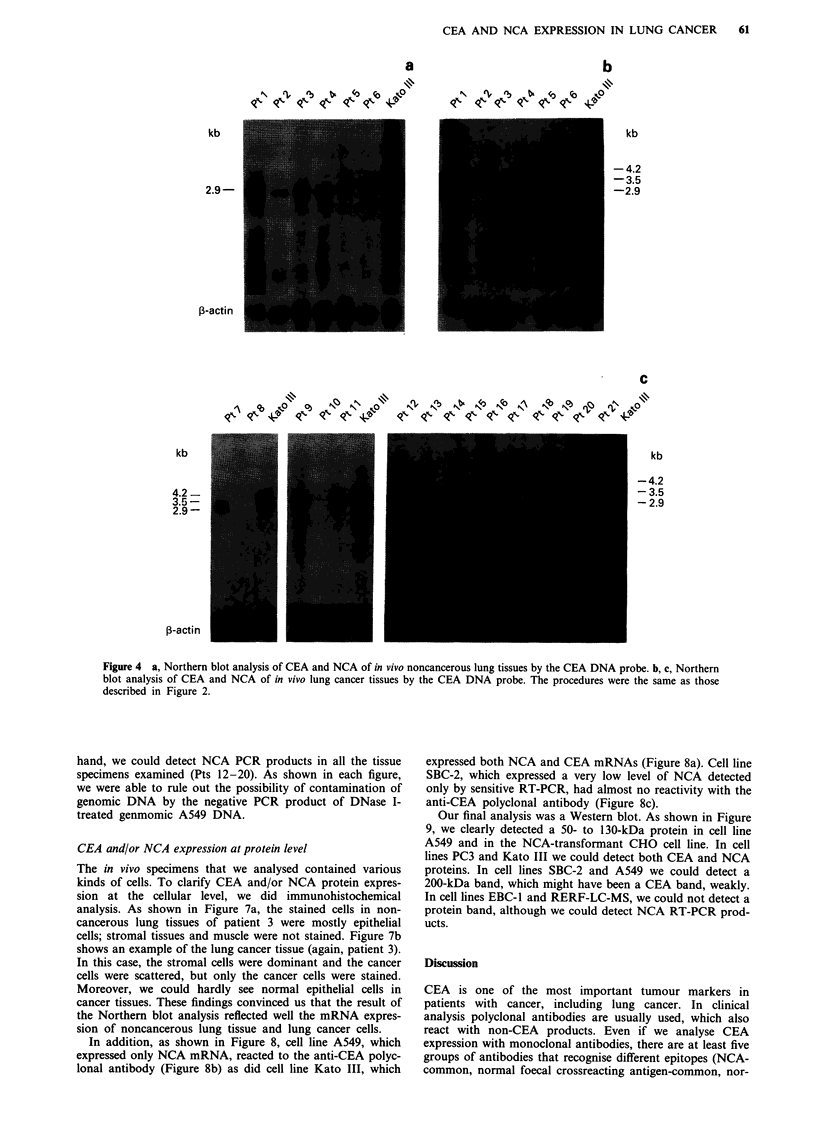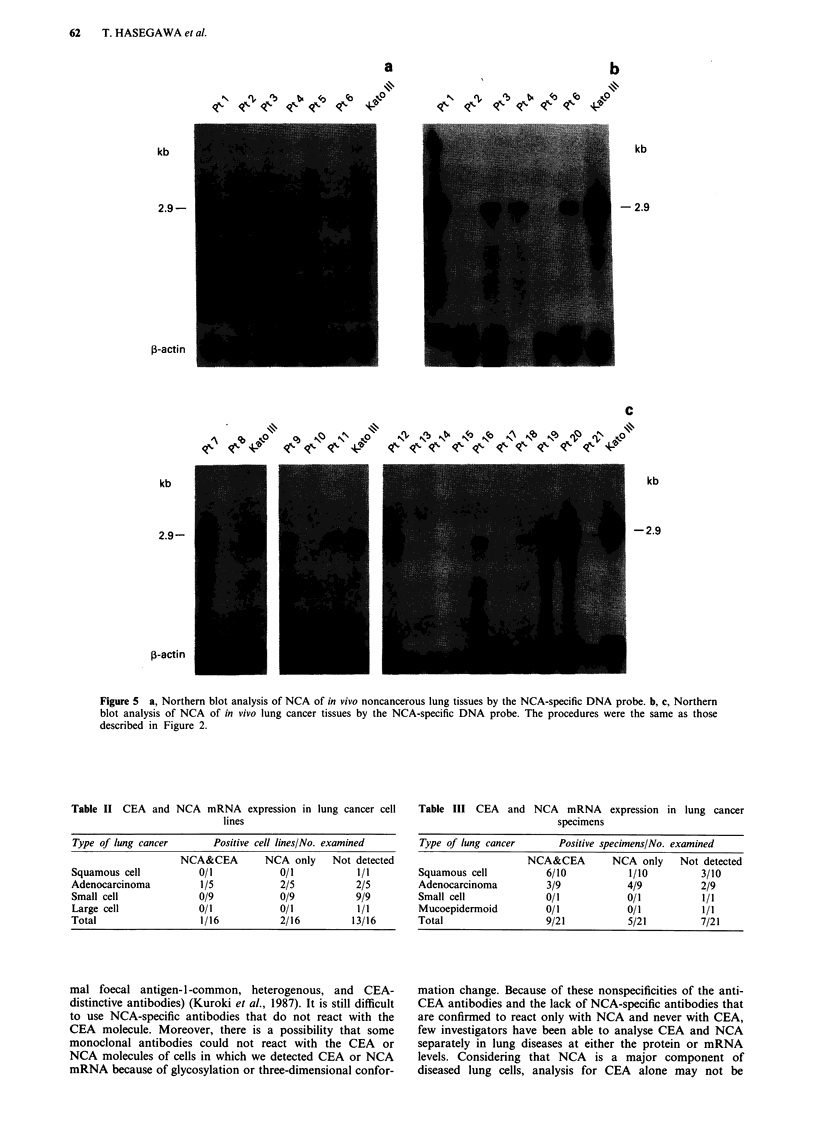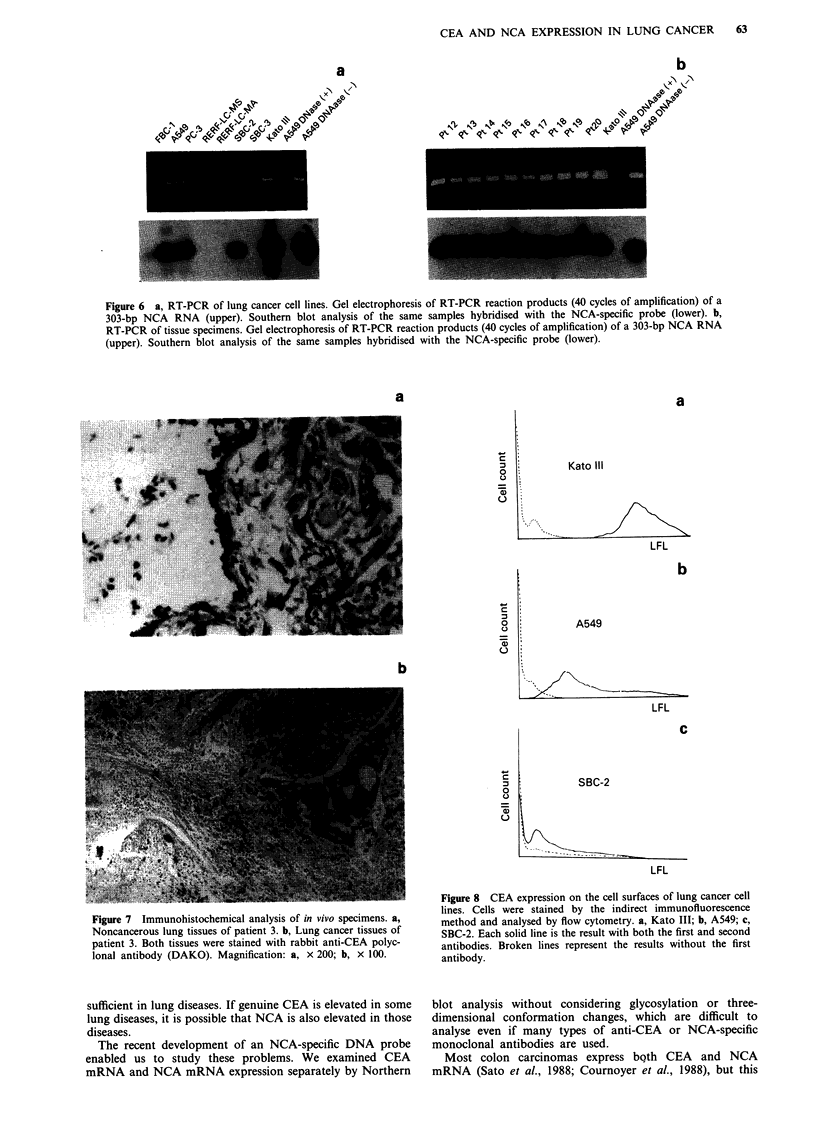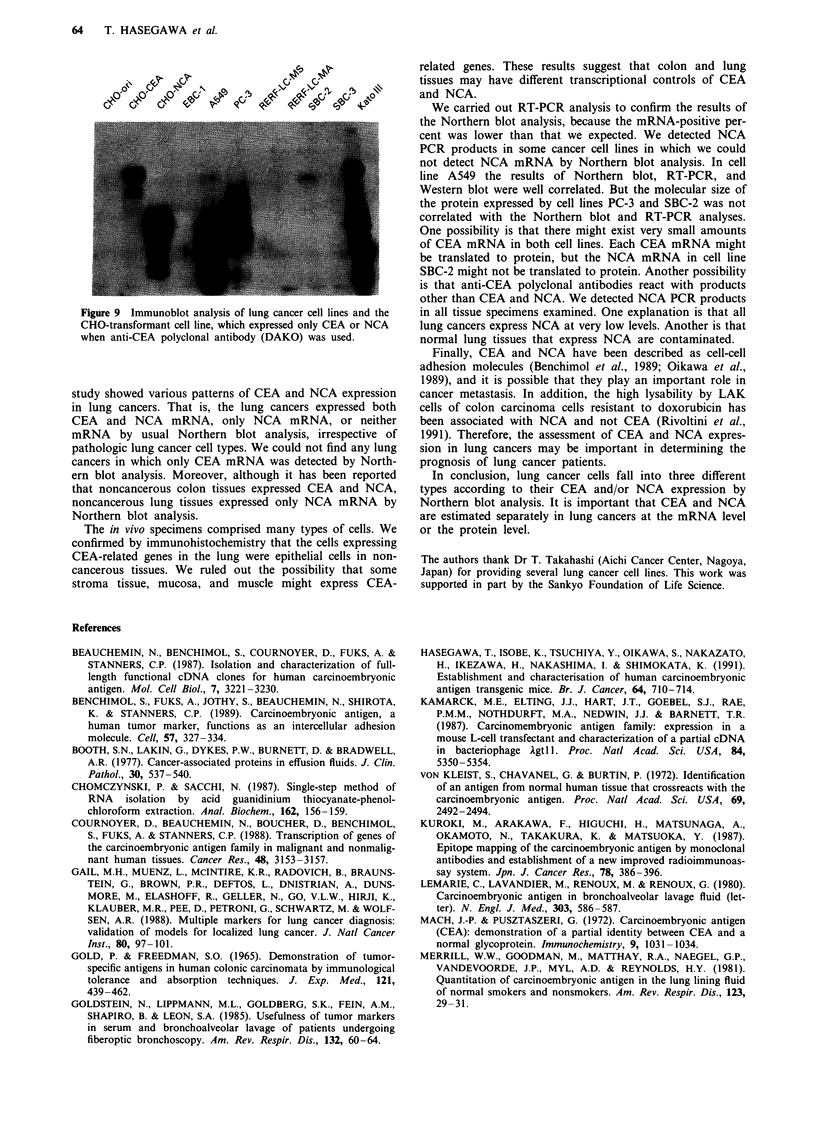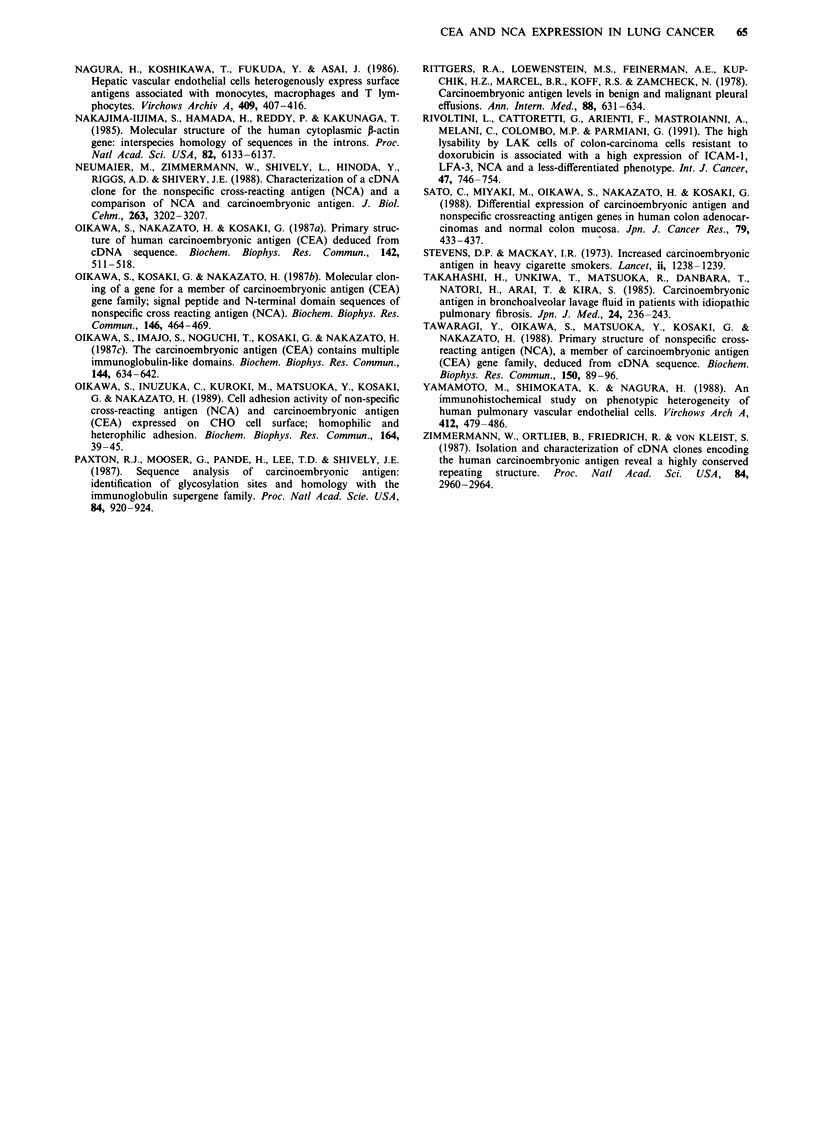# Nonspecific crossreacting antigen (NCA) is a major member of the carcinoembryonic antigen (CEA)-related gene family expressed in lung cancer.

**DOI:** 10.1038/bjc.1993.9

**Published:** 1993-01

**Authors:** T. Hasegawa, K. Isobe, Y. Tsuchiya, S. Oikawa, H. Nakazato, I. Nakashima, K. Shimokata

**Affiliations:** First Department of Internal Medicine, Nagoya University School of Medicine, Japan.

## Abstract

**Images:**


*Br. J. Cancer* (1993), **67**, 58–65 © Macmillan Press Ltd., 1993

### Nonspecific crossreacting antigen (NCA) is a major member of the carcinoembryonic antigen (CEA)-related gene family expressed in lung cancer

T. Hasegawa[1], K. Isobe[2], Y. Tsuchiya[1], S. Oikawa[3], H. Nakazato[3], I. Nakashima[2] & K. Shimokata[1]

[1]*First Department of Internal Medicine and* [2]*Department of Immunology, Nagoya University School of Medicine, 65 Tsurumai-cho, Showa-ku, Nagoya 466;* [3]*Suntory Institute for Biomedical Research, Shimamoto-cho, Mishimagun, Osaka 618, Japan.*

**Summary** Carcinoembryonic antigen (CEA) is one of the most important tumour markers in the management of human carcinoma, including lung cancer. So far, however, because of the nonspecificity of anti-CEA antibodies, it remains unclear whether the experimental measurements of CEA expression really reflect genuine CEA. In normal lung, nonspecific cross reacting antigen (NCA) has been described as a major component of CEA-related antigens. Recently isolated CEA and NCA cDNA clones enabled us to analyse CEA and NCA expression of *in vivo* tumour specimens and tumour cell lines at mRNA levels. NCA-specific mRNA (but not CEA-specific mRNA) was detected in all normal lung tissues examined. Of 21 lung cancer tissue specimens, nine expressed both NCA and CEA and five expressed only NCA. Of 16 tumour cell lines, two expressed only NCA and one expressed both NCA and CEA, although its level of CEA mRNA was weaker than that of NCA mRNA. Therefore, CEA-related mRNA expression was always accompanied by NCA mRNA expression; there were no cases of CEA mRNA expression alone. These findings suggest that NCA is a major member of the CEA-related gene family expressed in lung cancer.

Carcinoembryonic antigen (CEA), which was first described in 1965 as a colon tumour-specific antigen (Gold & Freedman, 1965), is an important clinical marker of lung cancer in serum (Gail *et al.*, 1988), pleural effusion (Booth *et al.*, 1977; Rittgers *et al.*, 1978) and bronchoalveolar lavage fluid (BALF) (Lemarie *et al.*, 1980; Goldstein *et al.*, 1985). The CEA concentrations in serum (Stevens & Mackay, 1973) and BALF (Merrill *et al.*, 1981) of smokers are higher than those of nonsmokers, and patients with idiopathic pulmonary fibrosis demonstrate a high level of CEA in BALF (Takahashi *et al.*, 1985). These analyses were made by anti-CEA antibodies. However, some anti-CEA antibodies, especially polyclonal antibodies, also react with nonspecific crossreacting antigen (NCA), a CEA-related glycoprotein and a major component of the CEA family in the lung (von Kleist *et al.*, 1972; Mach & Pusztaszeri, 1972). As far as we know, the target of these antibodies was so-called CEA, a compound of genuine CEA and NCA, and there has been little discussion about the relevance of NCA, especially at the mRNA level.

Recently, CEA (Oikawa *et al.*, 1987*a*; Zimmermann *et al.*, 1987; Kamarck *et al.*, 1987; Beauchemin *et al.*, 1987) and NCA (Oikawa *et al.*, 1987*b*; Tawaragi *et al.*, 1988; Neumaier *et al.*, 1988) cDNA clones were isolated and characterised. DNA analysis indicated that both of them are in the immunoglobulin superfamily (Oikawa *et al.*, 1987*c*; Paxton *et al.*, 1987). The development of CEA and NCA-specific DNA probes enabled us to discriminate the expression of CEA and NCA at the mRNA level. In this paper, we describe various patterns of CEA and NCA mRNA expression in lung cancer cell lines and *in vivo* specimens.

### Materials and methods

### *Tumour and nontumour specimens from lung cancer patients*

Twenty-one lung cancer tissue specimens and six normal lung tissue specimens were obtained from fresh surgical specimens at Nagoya University Hospital, Nagoya, Japan. Cancer types are listed in Table I.

### *Cell lines*

Lung cancer cell lines A549 (adenocarcinoma), RERF-LC-MA (small cell), RERF-LC-MS (adenocarcinoma), SBC-2 (small cell), SBC-3 (small cell), EBC-1 (squamous cell), and PC-3 (adenocarcinoma) and stomach cancer cell line Kato III were obtained from the Japanese Cancer Research Resources Bank. Lung cancer cell lines ACC-LC-170, 76, 67, 49, 177, 48 (small cell), SK-LC-4, 10 (adenocarcinoma) and Calu 6 (large cell) were obtained from Aichi Cancer Center (Dr T. Takahashi), Nagoya, Japan. Chinese hamster ovary (CHO) transformants expressing only CEA or NCA were established as previously described (Oikawa *et al.*, 1989). Each cell line was cultured in RPMI 1640 medium supplemented with 10% foetal calf serum.

### *DNA probe*

The CEA DNA probe, which hybridised with the 4.2 kb, 3.5 kb (CEA) and 2.9 kb (NCA) mRNAs, is a PvuII-digested DNA fragment of the pCEA 55-2 clone, CEA3 (Sato *et al.*, 1988) (Figure 1). The EcoRI-digested DNA fragment of the 3′-untranslated region of NCA clone 15 (Tawaragi *et al.*, 1988) was used as the NCA-specific DNA probe; it hybridised with the 2.9 kb (NCA) mRNA (Figure 1). Human $\beta$-actin probe (Nakajima-Iijima *et al.*, 1985) was used as an internal control.

**Table I** Characteristics of the patients

| *Tissue type* | *Pt. No.* |
|---|---|
| Squamous cell | Pt. 1, 5, 6, 7, 9, 10, 12, 13, 17, 18 |
| Adenocarcinoma | Pt. 3, 4, 8, 11, 15, 16, 19, 20, 21 |
| Small cell | Pt. 2 |
| Mucoepidermoid | Pt. 14 |
| Normal lung | Pt. 1, 2, 3, 4, 5, 6 |

Correspondence: K. Isobe.
Received 28 February 1992; and in revised form 17 August 1992.

**Figure 1** CEA and NCA DNA probes used in the present study and the position of the RT-PCR product.

*DNA primer*

A pair of DNA primers, 5′-GACAGCTTTTCCCAAGA-TGT-3′ (primer a) and 5′-AGTCTAGAAGTCCAACT-CTG-3′ (primer b), was used to amplify the 303-bp NCA-specific fragment of the 3′-untranslated region of NCA mRNA. The position of the primer is shown in Figure 1.

*RNA isolation and Northern blot analysis*

Total RNA was extracted from each cell line and lung tissue specimen following the methods described in the literature (Chomczynski & Sacchi, 1987). About 10 μg of each RNA preparation was electrophoresed on 1% agarose gels containing 1.1 M formaldehyde. The RNA was transferred to Hybond-N nylon membranes (Amersham). mRNA was detected by $^{32}$P-labelled probe (Multiprime labeling system, Amersham) by hybridisation for 18 h at 42°C in 5 × SSPE (1 × SSPE is composed of 0.18 M NaCl, 10 mM sodium phosphate (pH 7.7), and 1 mM EDTA), 5 × Denhardt's solution (1 × Denhart's solution is composed of 0.02% bovine serum albumin, 0.02% Ficoll, and 0.02% polyvinyl pyrollidone), 50% formamide, 0.1% sodium dodecyl sulphate (SDS), 50 μg ml$^{-1}$ heat-denatured salmon testis DNA, and radioactive probe. Membranes were washed twice for 15 min at 65°C in a solution containing 2 × SSC (1 × SSC is composed of 0.15 M NaCl and 15 mM sodium citrate) and 0.1% SDS, then once in 1 × SSC with 0.1% SDS for 30 min at 65°C, and finally twice for 15 min in 0.1% SSC with 0.1% SDS at room temperature. The membranes were then autoradiographed at − 70°C using Fuji RX film.

*Reverse transcriptase polymerase chain reaction (RT-PCR)*

The total RNA extracts of some lung cancer cell lines and *in vivo* lung cancer tissue specimens were first treated with DNase I (Takara) to eliminate contamination of the genomic DNA. After inactivation at 94°C for 5 min, RNA was then converted to cDNA by RT (RAV-2; Takara) at 42°C for 30 min with the primer b mentioned above. Again inactivated at 94°C for 5 min, the samples were amplified with primer a and *Taq* DNA polymerase (Promega). They were thermocycled at 94°C for 40 s, 55°C for 45 s, and 72°C for 1 min (40 cycles). PCR amplification products were evaluated by 3% agarose gel electrophoresis followed by Southern hybridisation using the NCA-specific probe. The hybridisation protocol was the same as that mentioned for Northern hybridisation.

*Immunohistochemical analysis*

The tissues were promptly fixed in periodate-lysine 4% paraformaldehyde for 6 h, washed in phosphate-buffered saline (PBS) containing increasing concentrations of sucrose, frozen in OCT compound (Lab Tek Products), and sectioned 6 μm thick on a cryostat. The sections were placed on egg-albumin coated slides and dried in air. Rabbit anti-human CEA polyclonal antibody (DAKO) was used as the first antibody. The goat anti-rabbit F(ab′)$_2$ fragment of immunoglobulin G (IgG) labelled with horseradish peroxidase (Organon Teknika) was used as the second antibody. Cryostat sections to be observed by light microscopy were treated with 100% methanol containing 0.03% hydrogen peroxidase to inactivate endogenous peroxidase. The indirect horseradish peroxidase-labelled antibody method was used for the immunological reaction, as previously described (Nagura *et al.*, 1986; Yamamoto *et al.*, 1988). Briefly, the procedure involved successive incubations with or without the first antibody in optimal dilutions for 12 h at 4°C and the second antibody for 6 h at 4°C. Sections were then treated with 0.25% diaminobenzidine (DAB) solution containing 0.01 M sodium azide and 0.01 M hydrogen peroxide and counterstained with methyl green.

*Immunofluorescence and laser flow cytometry*

Indirect immunofluorescence analysis was performed using anti-human CEA polyclonal antibody (DAKO) as the first antibody and fluorescein isothiocyanate (FITC)-conjugated anti-rabbit IgG goat antiserum (Organon Teknika) as the second antibody. The cell lines reacted with the first antibody for 1 h at 4°C. After three washings, cells were suspended in the medium containing the second antibody and incubated for 1 h at 4°C. The stained cells were resuspended in the medium after three washings and analysed on an EPICS profile flow cytometer (Coulter Corp.).

*Immunoblotting analysis*

About $10^7$ of some lung cancer cell lines were washed with PBS and homogenised. Extracts were sonicated, and 10 μl of each homogenate (about 10 μg) was resolved by 7.5% SDS-polyacrylamide gel electrophoresis (PAGE). The proteins were electrophoretically transferred to nitrocellulose membranes and visualised with rabbit anti-human CEA antibody (DAKO).

### Results

*Confirmation of specificity of NCA probe*

Northern hybridisation analysis was used to confirm the specificity of the NCA probe. We analysed the thymuses of CEA-transgenic mice established by us (Hasegawa *et al.*, 1991) and the Kato III tumour cell line. When we used the CEA probe, we could detect CEA mRNA in the transgenic mice's thymuses (data not shown) as mentioned before (Hasegawa *et al.*, 1991) and 4.2, 3.5, and 2.9 kb mRNAs in Kato III (Figure 2). However, when we used the NCA probe, we could not detect any mRNA in the CEA-transgenic mice's thymuses or in normal B6 mice's thymuses (Figure 3), but we could detect one band (2.9 kb) of mRNA in Kato III (Figure 3). The CEA probe we used hybridised not only with CEA but also with NCA mRNA, because the probe recognised the NCA coding region that has very high homology with the CEA coding region. From these results, we confirmed that the CEA probe hybridised with CEA (4.2, 3.5 kb) and NCA (2.9 kb) mRNAs and that the NCA probe hybridised only with NCA (2.9 kb) mRNA.

*Expression of NCA and CEA mRNAs in lung cancer cell lines*

Cell lines A549 and PC3 expressed 2.9 kb NCA mRNA, but not 4.2 or 3.5 kb CEA mRNAs when CEA (Figure 2) and NCA (Figure 3) probes were used. Cell line SK-LC-10 expressed 4.2, 3.5, and 2.9 kb mRNAs (Figure 2), although the 4.2 and 3.5 kb mRNAs were weaker than the 2.9 kb mRNA. The other cell lines expressed neither CEA nor NCA mRNA (Figures 2, 3).

*Expression of NCA and CEA mRNAs in tissue specimens*

We analysed the expression of NCA and CEA mRNAs in cancer tissues and adjacent noncancerous lung tissues. All noncancerous lung tissues examined expressed 2.9 kb mRNA

**Figure 2** Northern blot analysis of CEA and NCA in lung cancer cell lines by the CEA DNA probe. About 10 μg of total RNA was electrophoresed on a formaldehyde-agarose gel, transferred to a nylon membrane, and hybridised with the $^{32}P$-labelled PvuII fragment of CEA cDNA. After hybridisation, the filters were washed as described in the 'Materials and methods' section and autoradiographed. The same filters were rehybridised with human β-actin probe, and the results are shown at the bottom of the figure.

**Figure 3** Northern blot analysis of NCA in lung cancer cell lines by the NCA-specific DNA probe. The procedure was the same as that described in Figure 2.

but not 4.2 or 3.5 kb mRNAs (Figure 4a). Among 10 squamous cell lung cancer specimens, six expressed both NCA and CEA mRNAs (Figures 4b, c, Pts 1, 6, 9, 10, 12, 18), one expressed only NCA mRNA (Figure 4c, Pt 7), and the others expressed neither (Figures 4b, c, Pts 5, 13, 17). Of the adenocarcinomas, three expressed both NCA and CEA mRNAs (Figure 4c, Pts 11, 16, 19), four expressed only NCA mRNA (Figures 4b, c, Pts 3, 4, 20, 21), and the others expressed neither (Figure 4c, Pts 8, 15). One small-cell lung cancer specimen (Figure 4b, Pt2) and one mucoepidermoid lung cancer (Figure 4c, Pt 14) specimen expressed neither. From these results, we concluded that if the CEA-related genes were expressed in the lung, they always include NCA, but only occasionally include CEA. The NCA-specific probe confirmed these results (Figure 5). The analyses of cell lines and *in vivo* specimen are summarised in Tables II and III.

*Detection of NCA mRNA transcripts using RT-PCR*

To confirm the negative expression of NCA in lung cancer cell lines and tissue specimens, we carried out RT-PCR. As shown in Figure 6a, we could clearly detect NCA PCR products in cell lines A549, PC-3, and SBC-2 and weak bands in cell lines EBC-1 and RERF-LC-MS. We could not detect NCA-specific PCR products in cell lines SBC-3 and RERF-LC-MA, although we could observe a nonspecific DNA band by ethidium bromide staining. On the other

**Figure 4** **a**, Northern blot analysis of CEA and NCA of *in vivo* noncancerous lung tissues by the CEA DNA probe. **b**, **c**, Northern blot analysis of CEA and NCA of *in vivo* lung cancer tissues by the CEA DNA probe. The procedures were the same as those described in Figure 2.

hand, we could detect NCA PCR products in all the tissue specimens examined (Pts 12–20). As shown in each figure, we were able to rule out the possibility of contamination of genomic DNA by the negative PCR product of DNase I-treated genmomic A549 DNA.

### *CEA and/or NCA expression at protein level*

The *in vivo* specimens that we analysed contained various kinds of cells. To clarify CEA and/or NCA protein expression at the cellular level, we did immunohistochemical analysis. As shown in Figure 7a, the stained cells in noncancerous lung tissues of patient 3 were mostly epithelial cells; stromal tissues and muscle were not stained. Figure 7b shows an example of the lung cancer tissue (again, patient 3). In this case, the stromal cells were dominant and the cancer cells were scattered, but only the cancer cells were stained. Moreover, we could hardly see normal epithelial cells in cancer tissues. These findings convinced us that the result of the Northern blot analysis reflected well the mRNA expression of noncancerous lung tissue and lung cancer cells.

In addition, as shown in Figure 8, cell line A549, which expressed only NCA mRNA, reacted to the anti-CEA polyclonal antibody (Figure 8b) as did cell line Kato III, which expressed both NCA and CEA mRNAs (Figure 8a). Cell line SBC-2, which expressed a very low level of NCA detected only by sensitive RT-PCR, had almost no reactivity with the anti-CEA polyclonal antibody (Figure 8c).

Our final analysis was a Western blot. As shown in Figure 9, we clearly detected a 50- to 130-kDa protein in cell line A549 and in the NCA-transformant CHO cell line. In cell lines PC3 and Kato III we could detect both CEA and NCA proteins. In cell lines SBC-2 and A549 we could detect a 200-kDa band, which might have been a CEA band, weakly. In cell lines EBC-1 and RERF-LC-MS, we could not detect a protein band, although we could detect NCA RT-PCR products.

### Discussion

CEA is one of the most important tumour markers in patients with cancer, including lung cancer. In clinical analysis polyclonal antibodies are usually used, which also react with non-CEA products. Even if we analyse CEA expression with monoclonal antibodies, there are at least five groups of antibodies that recognise different epitopes (NCA-common, normal foecal crossreacting antigen-common, nor-

**Figure 5** **a**, Northern blot analysis of NCA of *in vivo* noncancerous lung tissues by the NCA-specific DNA probe. **b**, **c**, Northern blot analysis of NCA of *in vivo* lung cancer tissues by the NCA-specific DNA probe. The procedures were the same as those described in Figure 2.

**Table II** CEA and NCA mRNA expression in lung cancer cell lines

| *Type of lung cancer* | *Positive cell lines/No. examined* | | |
|---|---|---|---|
| | NCA&CEA | NCA only | Not detected |
| Squamous cell | 0/1 | 0/1 | 1/1 |
| Adenocarcinoma | 1/5 | 2/5 | 2/5 |
| Small cell | 0/9 | 0/9 | 9/9 |
| Large cell | 0/1 | 0/1 | 1/1 |
| Total | 1/16 | 2/16 | 13/16 |

**Table III** CEA and NCA mRNA expression in lung cancer specimens

| *Type of lung cancer* | *Positive specimens/No. examined* | | |
|---|---|---|---|
| | NCA&CEA | NCA only | Not detected |
| Squamous cell | 6/10 | 1/10 | 3/10 |
| Adenocarcinoma | 3/9 | 4/9 | 2/9 |
| Small cell | 0/1 | 0/1 | 1/1 |
| Mucoepidermoid | 0/1 | 0/1 | 1/1 |
| Total | 9/21 | 5/21 | 7/21 |

mal foecal antigen-1-common, heterogenous, and CEA-distinctive antibodies) (Kuroki *et al.*, 1987). It is still difficult to use NCA-specific antibodies that do not react with the CEA molecule. Moreover, there is a possibility that some monoclonal antibodies could not react with the CEA or NCA molecules of cells in which we detected CEA or NCA mRNA because of glycosylation or three-dimensional conformation change. Because of these nonspecificities of the anti-CEA antibodies and the lack of NCA-specific antibodies that are confirmed to react only with NCA and never with CEA, few investigators have been able to analyse CEA and NCA separately in lung diseases at either the protein or mRNA levels. Considering that NCA is a major component of diseased lung cells, analysis for CEA alone may not be

**Figure 6** **a**, RT-PCR of lung cancer cell lines. Gel electrophoresis of RT-PCR reaction products (40 cycles of amplification) of a 303-bp NCA RNA (upper). Southern blot analysis of the same samples hybridised with the NCA-specific probe (lower). **b**, RT-PCR of tissue specimens. Gel electrophoresis of RT-PCR reaction products (40 cycles of amplification) of a 303-bp NCA RNA (upper). Southern blot analysis of the same samples hybridised with the NCA-specific probe (lower).

**Figure 7** Immunohistochemical analysis of *in vivo* specimens. **a**, Noncancerous lung tissues of patient 3. **b**, Lung cancer tissues of patient 3. Both tissues were stained with rabbit anti-CEA polyclonal antibody (DAKO). Magnification: **a**, × 200; **b**, × 100.

**Figure 8** CEA expression on the cell surfaces of lung cancer cell lines. Cells were stained by the indirect immunofluorescence method and analysed by flow cytometry. **a**, Kato III; **b**, A549; **c**, SBC-2. Each solid line is the result with both the first and second antibodies. Broken lines represent the results without the first antibody.

sufficient in lung diseases. If genuine CEA is elevated in some lung diseases, it is possible that NCA is also elevated in those diseases.

The recent development of an NCA-specific DNA probe enabled us to study these problems. We examined CEA mRNA and NCA mRNA expression separately by Northern blot analysis without considering glycosylation or three-dimensional conformation changes, which are difficult to analyse even if many types of anti-CEA or NCA-specific monoclonal antibodies are used.

Most colon carcinomas express both CEA and NCA mRNA (Sato *et al.*, 1988; Cournoyer *et al.*, 1988), but this

**Figure 9** Immunoblot analysis of lung cancer cell lines and the CHO-transformant cell line, which expressed only CEA or NCA when anti-CEA polyclonal antibody (DAKO) was used.

study showed various patterns of CEA and NCA expression in lung cancers. That is, the lung cancers expressed both CEA and NCA mRNA, only NCA mRNA, or neither mRNA by usual Northern blot analysis, irrespective of pathologic lung cancer cell types. We could not find any lung cancers in which only CEA mRNA was detected by Northern blot analysis. Moreover, although it has been reported that noncancerous colon tissues expressed CEA and NCA, noncancerous lung tissues expressed only NCA mRNA by Northern blot analysis.

The *in vivo* specimens comprised many types of cells. We confirmed by immunohistochemistry that the cells expressing CEA-related genes in the lung were epithelial cells in noncancerous tissues. We ruled out the possibility that some stroma tissue, mucosa, and muscle might express CEA-related genes. These results suggest that colon and lung tissues may have different transcriptional controls of CEA and NCA.

We carried out RT-PCR analysis to confirm the results of the Northern blot analysis, because the mRNA-positive percent was lower than that we expected. We detected NCA PCR products in some cancer cell lines in which we could not detect NCA mRNA by Northern blot analysis. In cell line A549 the results of Northern blot, RT-PCR, and Western blot were well correlated. But the molecular size of the protein expressed by cell lines PC-3 and SBC-2 was not correlated with the Northern blot and RT-PCR analyses. One possibility is that there might exist very small amounts of CEA mRNA in both cell lines. Each CEA mRNA might be translated to protein, but the NCA mRNA in cell line SBC-2 might not be translated to protein. Another possibility is that anti-CEA polyclonal antibodies react with products other than CEA and NCA. We detected NCA PCR products in all tissue specimens examined. One explanation is that all lung cancers express NCA at very low levels. Another is that normal lung tissues that express NCA are contaminated.

Finally, CEA and NCA have been described as cell-cell adhesion molecules (Benchimol *et al.*, 1989; Oikawa *et al.*, 1989), and it is possible that they play an important role in cancer metastasis. In addition, the high lysability by LAK cells of colon carcinoma cells resistant to doxorubicin has been associated with NCA and not CEA (Rivoltini *et al.*, 1991). Therefore, the assessment of CEA and NCA expression in lung cancers may be important in determining the prognosis of lung cancer patients.

In conclusion, lung cancer cells fall into three different types according to their CEA and/or NCA expression by Northern blot analysis. It is important that CEA and NCA are estimated separately in lung cancers at the mRNA level or the protein level.

The authors thank Dr T. Takahashi (Aichi Cancer Center, Nagoya, Japan) for providing several lung cancer cell lines. This work was supported in part by the Sankyo Foundation of Life Science.

### References

BEAUCHEMIN, N., BENCHIMOL, S., COURNOYER, D., FUKS, A. & STANNERS, C.P. (1987). Isolation and characterization of full-length functional cDNA clones for human carcinoembryonic antigen. *Mol. Cell Biol.*, **7**, 3221–3230.

BENCHIMOL, S., FUKS, A., JOTHY, S., BEAUCHEMIN, N., SHIROTA, K. & STANNERS, C.P. (1989). Carcinoembryonic antigen, a human tumor marker, functions as an intercellular adhesion molecule. *Cell*, **57**, 327–334.

BOOTH, S.N., LAKIN, G., DYKES, P.W., BURNETT, D. & BRADWELL, A.R. (1977). Cancer-associated proteins in effusion fluids. *J. Clin. Pathol.*, **30**, 537–540.

CHOMCZYNSKI, P. & SACCHI, N. (1987). Single-step method of RNA isolation by acid guanidinium thiocyanate-phenol-chloroform extraction. *Anal. Biochem.*, **162**, 156–159.

COURNOYER, D., BEAUCHEMIN, N., BOUCHER, D., BENCHIMOL, S., FUKS, A. & STANNERS, C.P. (1988). Transcription of genes of the carcinoembryonic antigen family in malignant and nonmalignant human tissues. *Cancer Res.*, **48**, 3153–3157.

GAIL, M.H., MUENZ, L., MCINTIRE, K.R., RADOVICH, B., BRAUNSTEIN, G., BROWN, P.R., DEFTOS, L., DNISTRIAN, A., DUNSMORE, M., ELASHOFF, R., GELLER, N., GO, V.L.W., HIRJI, K., KLAUBER, M.R., PEE, D., PETRONI, G., SCHWARTZ, M. & WOLFSEN, A.R. (1988). Multiple markers for lung cancer diagnosis: validation of models for localized lung cancer. *J. Natl Cancer Inst.*, **80**, 97–101.

GOLD, P. & FREEDMAN, S.O. (1965). Demonstration of tumor-specific antigens in human colonic carcinomata by immunological tolerance and absorption techniques. *J. Exp. Med.*, **121**, 439–462.

GOLDSTEIN, N., LIPPMANN, M.L., GOLDBERG, S.K., FEIN, A.M., SHAPIRO, B. & LEON, S.A. (1985). Usefulness of tumor markers in serum and bronchoalveolar lavage of patients undergoing fiberoptic bronchoscopy. *Am. Rev. Respir. Dis.*, **132**, 60–64.

HASEGAWA, T., ISOBE, K., TSUCHIYA, Y., OIKAWA, S., NAKAZATO, H., IKEZAWA, H., NAKASHIMA, I. & SHIMOKATA, K. (1991). Establishment and characterisation of human carcinoembryonic antigen transgenic mice. *Br. J. Cancer*, **64**, 710–714.

KAMARCK, M.E., ELTING, J.J., HART, J.T., GOEBEL, S.J., RAE, P.M.M., NOTHDURFT, M.A., NEDWIN, J.J. & BARNETT, T.R. (1987). Carcinomembryonic antigen family: expression in a mouse L-cell transfectant and characterization of a partial cDNA in bacteriophage λgt11. *Proc. Natl Acad. Sci. USA*, **84**, 5350–5354.

VON KLEIST, S., CHAVANEL, G. & BURTIN, P. (1972). Identification of an antigen from normal human tissue that crossreacts with the carcinoembryonic antigen. *Proc. Natl Acad. Sci. USA*, **69**, 2492–2494.

KUROKI, M., ARAKAWA, F., HIGUCHI, H., MATSUNAGA, A., OKAMOTO, N., TAKAKURA, K. & MATSUOKA, Y. (1987). Epitope mapping of the carcinoembryonic antigen by monoclonal antibodies and establishment of a new improved radioimmunoassay system. *Jpn. J. Cancer Res.*, **78**, 386–396.

LEMARIE, C., LAVANDIER, M., RENOUX, M. & RENOUX, G. (1980). Carcinoembryonic antigen in bronchoalveolar lavage fluid (letter). *N. Engl. J. Med.*, **303**, 586–587.

MACH, J.-P. & PUSZTASZERI, G. (1972). Carcinoembryonic antigen (CEA): demonstration of a partial identity between CEA and a normal glycoprotein. *Immunochemistry*, **9**, 1031–1034.

MERRILL, W.W., GOODMAN, M., MATTHAY, R.A., NAEGEL, G.P., VANDEVOORDE, J.P., MYL, A.D. & REYNOLDS, H.Y. (1981). Quantitation of carcinoembryonic antigen in the lung lining fluid of normal smokers and nonsmokers. *Am. Rev. Respir. Dis.*, **123**, 29–31.

NAGURA, H., KOSHIKAWA, T., FUKUDA, Y. & ASAI, J. (1986). Hepatic vascular endothelial cells heterogenously express surface antigens associated with monocytes, macrophages and T lymphocytes. *Virchows Archiv A*, **409**, 407–416.

NAKAJIMA-IIJIMA, S., HAMADA, H., REDDY, P. & KAKUNAGA, T. (1985). Molecular structure of the human cytoplasmic β-actin gene: interspecies homology of sequences in the introns. *Proc. Natl Acad. Sci. USA*, **82**, 6133–6137.

NEUMAIER, M., ZIMMERMANN, W., SHIVELY, L., HINODA, Y., RIGGS, A.D. & SHIVERY, J.E. (1988). Characterization of a cDNA clone for the nonspecific cross-reacting antigen (NCA) and a comparison of NCA and carcinoembryonic antigen. *J. Biol. Cehm.*, **263**, 3202–3207.

OIKAWA, S., NAKAZATO, H. & KOSAKI, G. (1987*a*). Primary structure of human carcinoembryonic antigen (CEA) deduced from cDNA sequence. *Biochem. Biophys. Res. Commun.*, **142**, 511–518.

OIKAWA, S., KOSAKI, G. & NAKAZATO, H. (1987*b*). Molecular cloning of a gene for a member of carcinoembryonic antigen (CEA) gene family; signal peptide and N-terminal domain sequences of nonspecific cross reacting antigen (NCA). *Biochem. Biophys. Res. Commun.*, **146**, 464–469.

OIKAWA, S., IMAJO, S., NOGUCHI, T., KOSAKI, G. & NAKAZATO, H. (1987*c*). The carcinoembryonic antigen (CEA) contains multiple immunoglobulin-like domains. *Biochem. Biophys. Res. Commun.*, **144**, 634–642.

OIKAWA, S., INUZUKA, C., KUROKI, M., MATSUOKA, Y., KOSAKI, G. & NAKAZATO, H. (1989). Cell adhesion activity of non-specific cross-reacting antigen (NCA) and carcinoembryonic antigen (CEA) expressed on CHO cell surface; homophilic and heterophilic adhesion. *Biochem. Biophys. Res. Commun.*, **164**, 39–45.

PAXTON, R.J., MOOSER, G., PANDE, H., LEE, T.D. & SHIVELY, J.E. (1987). Sequence analysis of carcinoembryonic antigen: identification of glycosylation sites and homology with the immunoglobulin supergene family. *Proc. Natl Acad. Scie. USA*, **84**, 920–924.

RITTGERS, R.A., LOEWENSTEIN, M.S., FEINERMAN, A.E., KUPCHIK, H.Z., MARCEL, B.R., KOFF, R.S. & ZAMCHECK, N. (1978). Carcinoembryonic antigen levels in benign and malignant pleural effusions. *Ann. Intern. Med.*, **88**, 631–634.

RIVOLTINI, L., CATTORETTI, G., ARIENTI, F., MASTROIANNI, A., MELANI, C., COLOMBO, M.P. & PARMIANI, G. (1991). The high lysability by LAK cells of colon-carcinoma cells resistant to doxorubicin is associated with a high expression of ICAM-1, LFA-3, NCA and a less-differentiated phenotype. *Int. J. Cancer*, **47**, 746–754.

SATO, C., MIYAKI, M., OIKAWA, S., NAKAZATO, H. & KOSAKI, G. (1988). Differential expression of carcinoembryonic antigen and nonspecific crossreacting antigen genes in human colon adenocarcinomas and normal colon mucosa. *Jpn. J. Cancer Res.*, **79**, 433–437.

STEVENS, D.P. & MACKAY, I.R. (1973). Increased carcinoembryonic antigen in heavy cigarette smokers. *Lancet*, **ii**, 1238–1239.

TAKAHASHI, H., UNKIWA, T., MATSUOKA, R., DANBARA, T., NATORI, H., ARAI, T. & KIRA, S. (1985). Carcinoembryonic antigen in bronchoalveolar lavage fluid in patients with idiopathic pulmonary fibrosis. *Jpn. J. Med.*, **24**, 236–243.

TAWARAGI, Y., OIKAWA, S., MATSUOKA, Y., KOSAKI, G. & NAKAZATO, H. (1988). Primary structure of nonspecific cross-reacting antigen (NCA), a member of carcinoembryonic antigen (CEA) gene family, deduced from cDNA sequence. *Biochem. Biophys. Res. Commun.*, **150**, 89–96.

YAMAMOTO, M., SHIMOKATA, K. & NAGURA, H. (1988). An immunohistochemical study on phenotypic heterogeneity of human pulmonary vascular endothelial cells. *Virchows Arch A*, **412**, 479–486.

ZIMMERMANN, W., ORTLIEB, B., FRIEDRICH, R. & VON KLEIST, S. (1987). Isolation and characterization of cDNA clones encoding the human carcinoembryonic antigen reveal a highly conserved repeating structure. *Proc. Natl Acad. Sci. USA*, **84**, 2960–2964.